# Does the microbiome and virome contribute to myalgic encephalomyelitis/chronic fatigue syndrome?

**DOI:** 10.1042/CS20171330

**Published:** 2018-03-09

**Authors:** Fiona Newberry, Shen-Yuan Hsieh, Tom Wileman, Simon R. Carding

**Affiliations:** 1 Norwich Medical School, University of East Anglia, Norwich NR4 7TJ, U.K.; 2 The Gut Health and Food Safety Research Programme, The Quadram Institute, Norwich Research Park, Norwich, U.K.

**Keywords:** Myalgic Encephalomyelitis/Chronic Fatigue Syndrome, microbiome, Virome

## Abstract

Myalgic encephalomyelitis (ME)/chronic fatigue syndrome (CFS) (ME/CFS) is a disabling and debilitating disease of unknown aetiology. It is a heterogeneous disease characterized by various inflammatory, immune, viral, neurological and endocrine symptoms. Several microbiome studies have described alterations in the bacterial component of the microbiome (dysbiosis) consistent with a possible role in disease development. However, in focusing on the bacterial components of the microbiome, these studies have neglected the viral constituent known as the virome. Viruses, particularly those infecting bacteria (bacteriophages), have the potential to alter the function and structure of the microbiome via gene transfer and host lysis. Viral-induced microbiome changes can directly and indirectly influence host health and disease. The contribution of viruses towards disease pathogenesis is therefore an important area for research in ME/CFS. Recent advancements in sequencing technology and bioinformatics now allow more comprehensive and inclusive investigations of human microbiomes. However, as the number of microbiome studies increases, the need for greater consistency in study design and analysis also increases. Comparisons between different ME/CFS microbiome studies are difficult because of differences in patient selection and diagnosis criteria, sample processing, genome sequencing and downstream bioinformatics analysis. It is therefore important that microbiome studies adopt robust, reproducible and consistent study design to enable more reliable and valid comparisons and conclusions to be made between studies. This article provides a comprehensive review of the current evidence supporting microbiome alterations in ME/CFS patients. Additionally, the pitfalls and challenges associated with microbiome studies are discussed.

## Graphical abstract

**Graphical Abstract:**
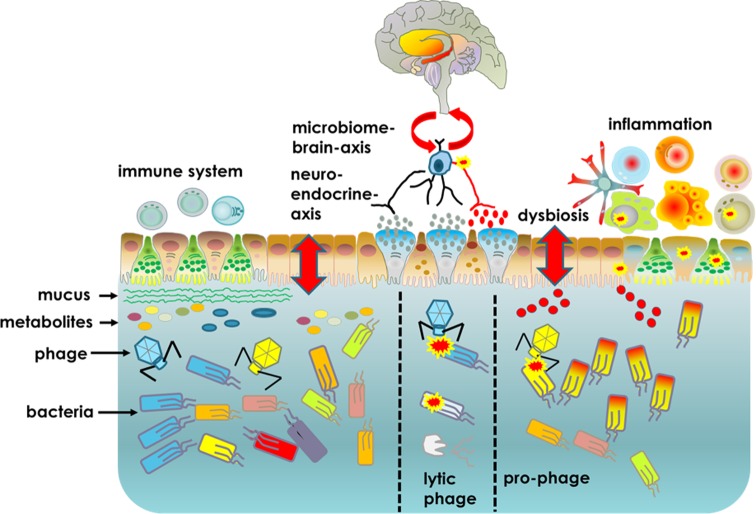
The gut lumen contains large numbers of different bacteria which generate metabolites that together help maintain mucosal barrier function and reduce inflammation. The gut lining also contains enteroendocrine cells that secrete hormones which influence the enteric nervous system. These cells sense metabolites in the gut creating a microbiome–brain axis. Each bacterium can be infected by a bacteriophage and this can influence the microbiota. In this example, a blue bacteria is infected by a blue lytic phage and killed. The blue bacteria and its beneficial metabolites are lost from the microbiota. A second yellow bacteria is infected by a yellow prophage which integrates into the bacterial genome giving a selective advantage. Yellow bacteria and their harmful metabolites (red) predominate leading to dysbiosis, inflammation and altered enteric nervous system.

## What is the microbiome?

Virtually every surface of the human body is colonized by vast populations of microbes, including prokaryotes, archaea, viruses, fungi and unicellular eukaryotes [1–3]. Bacteria of the phyla Bacteriodetes and Firmicutes dominate the diverse and complex intestinal bacteriome of most animals [4]. Microbial colonization begins rapidly at birth when the infant is first exposed to microbes in its immediate environment. The microbiome increases in diversity during the first 2–4 years of life in response to various hosts (i.e. genetics), and environmental factors including diet, lifestyle and behaviour [5–7]. It is believed that the early colonizers of the infant intestine play a key role in laying the foundations for the development of the complex and diverse adult microbiome and lifelong health [8]. In recent years, the role of microbiome in health of the host and its contribution to disease development has emerged [9–11]. It contributes to various body systems including immunity, metabolism, neurological signalling and homeostasis [12,13]. Describing the microbiome in detail is beyond the scope of this article, however several excellent review papers have been published recently [7,14–17].

### The neglected virome

The vast majority of microbiome research has to date focused on its bacterial component, largely neglecting the other organisms. However, the influence of these lesser studied organisms, such as viruses are just beginning to be understood, thanks primarily to recent advancements in sequencing technology and bioinformatics capability (Table 1) [18].

**Table 1:** Overview of important faecal virome studies in health and disease

Year	Study participants	Comments	Reference
2003	Healthy adults	First virome metagenomics study	[168]
2006	Healthy adults	Plant RNA viruses contribute towards virome	[169]
2008	Infants	Virome establishment begins within 1 week of birth	[21]
2011	Healthy adults	Diversity and abundance of ssDNA viruses	[170]
2011	Monozygotic twins and mothers	Virome is individualized and highly stable	[22]
2011	Healthy adults	Virome is influenced by diet	[157]
2012	Healthy adults	Hypervariation driven by unique reverse transcriptase based mechanism	[171]
2013	Healthy adult	Virome is relatively stable; 80% of virome remained through 2.5-year study	[172]
2013	Pediatric CD patients	CD patients exhibited higher bacteriophage levels than controls	[49]
2013	CD patients	Similar results as above; results depend on interpretation of data	[173]
2015	Infants	Longitudinal study of virome establishment in infant twins	[174]
2015	Malnourished Malawian twins	Virome establishment affected by severe malnourishment	[178]
2015	IBD patients	Virome in IBD patients	[51]
2015	IBD patients	Increase in phage-richness abundance compared with healthy controls	[175]
2015	CD patients	Alterations in virome according to disease status and therapy	[58]

Abbreviations: CD, Crohn’s disease; IBD; inflammatory bowel disease.

**Figure 1:**
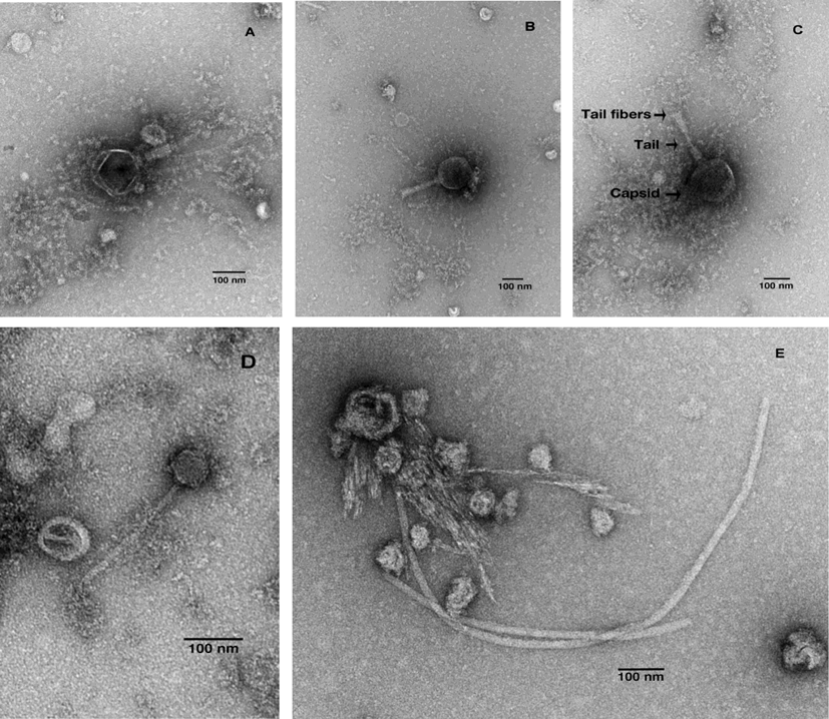
TEM images of Caudovirales from faecal water (**A**–**C**) Myoviridae and (**D,E**) Siphoviridae. Imaging completed by S.-Y.H. and K.C.

It is estimated that there are 10^31^ different DNA and RNA viruses on the planet; many of which remain undiscovered [19]. This collection of viruses (dsDNA, ssDNA, dsRNA and ssRNA) within an ecosystem is defined as the virome [20]. Similar to the bacteriome, the intestinal virome is established from birth and increases in diversity/complexity with age [21]. A large proportion of this complex environment consists of prokaryotic viruses (bacteriophage); with archaea-, human-, plant- and amoeba-infecting viruses found at lower frequencies [20]. The tailed, dsDNA viruses of the Order Caudovirales (Siphoviridae, Myoviridae, Podoviridae) dominate the bacteriophage portion of the virome (Figure 1) [22].

## The microbiota in health and disease

The importance of the intestinal microbiome in maintaining health is an emerging research topic with advances in high-throughput sequencing technology allowing the identification and characterization of microbes that contribute to host health [10,11]. The microbiota has been implicated in immunomodulation, pathogen resistance, maintenance of intestine structure/function and nutrition and host metabolism [12,13]. It provides the host with a physical barrier to pathogen invasion and infection by, for example competitive exclusion and competing for nutrients, occupation of attachment sites and production of antimicrobial proteins [23–26]. Importantly, various microbiome members have been found to contribute to the intestinal metabolome, through for example vitamin synthesis, bile salt metabolism and xenobiotic degradation [27]. There is bidirectional communication between the microbiome and the local host immune system [28]. The immune system influences the composition of the microbiota and gut microbes and their products (e.g. metabolites and microbe associated molecular pattern (MAMPs) molecules) direct immune maturation and the development and possibly maintenance of immune (microbial) tolerance and homoeostasis [29,30].

There is increasing evidence that an imbalance of the intestinal microbiota (dysbiosis) may contribute to the pathogenesis of diseases affecting the gastrointestinal (GI) tract and other organ systems. Dysbiosis is characterized by a detrimental alteration of intestine microbial populations and ecology that can result in the growth and expansion of pathogenic microbes (pathobionts) and the production of factors toxic or harmful to host cells. These alterations are normally held in check by an intact microbiome but dysbiosis can result in the development and/or maintenance of chronic inflammatory infections caused by *Clostridium difficile* and *Helicobacter pylori*, metabolic syndrome and obesity, colorectal cancer, irritable bowel syndrome (IBS) and inflammatory bowel disease (IBD) [14,31–34]. The stability of the microbiome is largely influenced by age, behaviour and lifestyle [12,35,36]. It has been hypothesized that intestinal microbial dysbiosis can lead to an imbalance in the immune system, resulting in diseases such as IBD, common variable immunodeficiency and rheumatoid arthritis [37,38]. However, to understand the significance of dysbiosis, it is necessary to establish if microbiome alterations cause, follow, precede or simply correlate with disease onset. Dysbiosis can be precipitated by drugs and medications (i.e. antibiotics), immune dysregulation, age-associated reduction in microbiota diversity, colonization by pathogenic microbes, stress and changes in diet [36,38–41]. The precise trigger or cause of dysbiosis in any disease has yet to be established but is likely to be multifactorial.

### The virome in health

Viruses utilize a lytic or lysogenic life cycle. In the lytic life cycle, infected host cells are destroyed during viral replication whereas in the lysogenic life cycle the virus integrates into the host chromosome as a prophage. Lytic phages can have both narrow or broad host ranges, and lysogenic phages can be converted into a lytic cycle in response to environmental stressors such as antibiotics [20]. Lytic phages can alter the microbiome by killing bacterial hosts, providing a competitive growth advantage to bacteria resistant to phages. Prophages encode mobile genetic elements which contribute to horizontal gene transfer between bacteria altering antibiotic resistance, virulence or metabolic pathways [42]. This can provide a competitive advantage by allowing bacteria to metabolize new nutrient sources or acquire antibiotic resistance [43–45]. Temperate phages, able to perform lysogenic or lytic cycle, have been shown to influence the dynamics of biofilms and dispersal by a number of important pathogens such as *Pseudomonas aeruginosa* (opportunistic pathogen), *Streptococcus pneumoniae* (e.g. pneumonia) and *Bacillus anthracis* (e.g. anthrax) [46–48]. For example, the presence of lysogenic phages Bcp1, Wip1, Wip4 and Frp2 in *B. anthracis* results in the formation of a durable, complex and viable biofilm; allowing prolonged survival of the bacterium [48]. There is a constant shift of phages between lytic and lygosenic forms that is presumed to contribute to microbiome homoeostasis and that a differential spatial distribution of phages is correlated with health.

### The virome in disease

**Figure 2:**
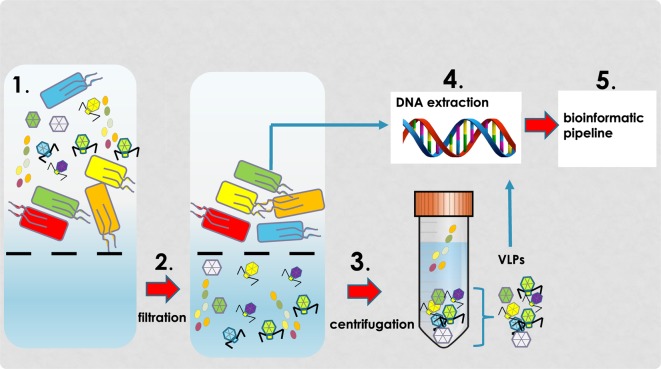
VLP isolation protocol Overview of VLP isolation protocol involving filtration (**1,2**) and centrifugation (**3**). Following isolation of concentration VLPs, DNA is extracted (**4**), sequencing and (**5**) bioinformatic tools applied to determine virome community composition.

Alterations in the virome have been implicated as sources of intestinal microbial (prokaryotic) dysbiosis for several different diseases [49–52]. Prophage induction in response to various environmental stressors can induce ‘community shuffling’ which alters the ratio of symbionts to pathobionts creating an imbalance within microbial communities that can lead to occupation of symbiont niches by pathobionts [42]. These events provide an explanation for the raised number of virus-like particles (VLPs, see Figure 2) as well as microbial population shifts in patients with GI-related disorders. Of note, an experimental model of *Salmonella typhimurium* diarrhoea has shown that inflammation increases lysogenic conversion of prophages [53].

The involvement of lytic phages in disease pathogenesis has been demonstrated through studying prophages that encode virulence factors (e.g. Shiga toxin) [54]. In the healthy intestine, toxin gene expression is silent in lysogenic phages that infect *Escherichia coli*. However, dysbiosis is accompanied by induction of prophages and activation of Shiga toxin genes resulting in release of the toxin into the intestine [55]. Additionally, *in vitro* experiments have shown phages can transmigrate across epithelial barrier cells. A recent study suggests that intestinal phages can interact directly with eukaryotic cells outside the GI tract; likely contributing to human health and immunity [56].

Although the virome is suspected to play a role in disease, relatively few studies have been undertaken with most studies focusing on IBD and HIV [49,50,52,57]. In one study, Crohn’s disease (CD) patients were shown to have a higher number of VLPs, which provide a crude estimate of phage numbers, in colonic biopsies compared with healthy controls. Patients with ulcerated mucosa had significantly fewer VLPs than non-ulcerated mucosa. The authors hypothesized that viruses had higher survival rates or a higher frequency of viruses in non-ulcerated areas. Based upon these findings, it was proposed that phages play an indirect role in the immune dysregulation evident in CD patients through microbiome alterations [57]. A later study also described differences in the virome in CD patients according to their disease status (newly diagnosed, active onset, active presurgery) and therapy. Newly diagnosed patients had a higher viral diversity in faecal and biopsy samples than those with active disease. Additionally, there were significant differences in virome diversity between patients on immunosuppressive therapy, steroids, combination therapy or no therapy. The clinical relevance of these results is unknown although more detailed studies of the alteration in virome composition in IBD patients is warranted [58]. Alterations in the enteric virome have also been reported in children susceptible to developing type 1 diabetes (T1D), prior to disease onset. Importantly, a disease-specific sequencing ‘fingerprint’ was identified in children susceptible to T1D that went on to develop the disease, compared with children that did not develop the disease. The present study reported that specific components of the virome were both directly and inversely associated with the development of T1D in these patients [59].

Additionally, a bidirectional communication network between the intestinal and central nervous system (the gut–brain–axis) is gaining research focus [60]. Its’ role is to monitor and integrate intestine functions as well as linking emotional and cognitive centres of the brain with peripheral intestinal functions and mechanisms such as immune system activation, intestinal permeability, enteric reflex, pain perception and enteroendocrine signalling [61]. Both clinical and experimental evidence suggest that the enteric microbiota has an important impact on communication pathways between the intestine and brain, also known as the gut–microbiome–brain axis. The microbiome can interact locally with intestinal cells and enteric nervous system, but also have indirect interactions with the CNS through neuroendocrine and metabolic pathways. Therefore, significant alterations in the resident microbiota or their metabolites might have a direct effect on the host nervous system and lead to neurological pathologies [62]. For example, changes in the microbiome have been associated with autism, depression, schizophrenia, Alzheimer’s disease and Parkinson’s disease [63–67].

Our own research is focused on developing a mechanistic understanding of the intestine–microbiome–brain axis and the GI tract microbiome in the pathogenesis of the neurological disorder, myalgic encephalomyelitis (ME)/chronic fatigue syndrome (CFS) (ME/CFS).

## ME/CFS

### Historical perspective

The causative factor(s) of ME/CFS remain elusive resulting in a lack of robust diagnostics and effective treatments [68,69]. The disease onset and progression varies from patient to patient with the onset normally associated with an acute flu-like viral infection, which is either gradual or rapid [68]. Approximately 25% of patients become house- or bed-bound with less than 10% returning to predisease levels of function [70]. The socioeconomic burden of ME/CFS is significant and estimated to be between $17 and $24 billion per annum. This considerable cost is due to direct and indirect effects of the illness, such as healthcare and loss of work for patient and/or family carers [71].

The heterogenic nature of ME/CFS suggests a multifactorial and self-sustaining disorder [72]. Several theories have been proposed including mitochondrial dysfunction, viral infection and autoimmunity [68,73,74]. Important clues for the involvement of (viral) infections in the aetiology of ME/CFS can be obtained from historical reports of epidemic or sporadic outbreaks of cases; the first of which was reported in 1934 in a suspected epidemic of poliomyelitis in Los Angeles, California [75,76]. The inconsistent disease pattern observed in patients led doctors to classify this epidemic as atypical; differing from polio cases endemic at the time by the lack of flaccid paralysis, which normally defines poliomyelitis [77]. Additionally, the affected cases were mainly older children and young adults compared with polio which affected infants and children of less than 5 years of age [78]. The disease at the onset consisted of an acute upper respiratory tract infection accompanied by muscle weakness, fever, pain, malaise and photophobia. The patients reported recurrence of fever and other symptoms during recovery, which were at a greater incidence than those in typical epidemic poliomyelitis [76].

A similar apparent epidemic of poliomyelitis appeared in Akureyri, Iceland between 1948 and 1949. There were striking similarities between this outbreak of atypical poliomyelitis and the one recorded in Los Angeles in 1934, including both overlapping symptoms and occurrence of relapse. This disease was named Iceland (or Akureyri) disease [79]. Sixty-one other outbreaks of a similar disease were reported worldwide between 1934 and 1990 [75]. The most significant outbreak was in 1955 at the Royal Free Hospital in London, where 292 hospital staff were affected by the illness. The disease when fully developed showed features of a generalized infection with involvement of the lymphoreticular system, and widespread involvement of the central nervous system. The mysterious polio-like illness (including the disease at the Royal Free Hospital) was renamed ME and later extended to CFS (ME/CFS) to include a seemingly identical disease [80,81].

### What is ME/CFS?

In both historical and current cases of ME/CFS persistent fatigue is the dominant and defining symptom, which is accompanied by a range of heterogeneous symptoms that are universally present in all the patients. It is classified by the World Healthy Organization International Classification of Diseases (ICD-10) as a neurological disorder (WHO Reference 93.3). Patients often report delayed exacerbation of symptoms following mental or physical exertion and daily or weekly variations in symptom severity that have a significant impact on day-to-day living [69,82]. A standardized criterion for ME/CFS is urgently needed, with diagnosis relying heavily upon clinical observations and by exclusion of other disorders. This situation is further complicated by the use of different diagnostic criteria within the same country and between different countries. As a result, it is can take several years for sufferers to receive a diagnosis [83–85]. To date, an effective treatment for ME/CFS does not exist, with current treatments aimed at alleviating symptoms [86].

### An intestinal origin for ME/CFS

The co-morbidity of ME/CFS and GI symptoms is well documented, with one study reporting 92% of patients exhibiting IBS [87]. Additional studies have reported increased mucosal and systemic levels of pro-inflammatory cytokines such as IL-6, IL-8, IL-1β and TNFα in patients with coexistent IBS [88,89]. The significant GI symptoms often experienced by ME/CFS patients has led researchers, including ourselves, to investigate the microbiome in these patients. Several studies have reported significant changes in microbiota composition of ME/CFS patients compared with controls [90–92]. However, ME/CFS microbiome studies to date have largely focused on alterations in bacterial populations. The advancement in sequencing technology and emerging influence of the virome on human health has enabled studies of the virome of ME/CFS [18].

### Intestinal microbiome and ME/CFS

We completed a literature search to determine the extent of microbiome research in ME/CFS using the following search terms: ‘Myalgic encephalomyelitis’, ‘Chronic Fatigue Syndrome’, ‘CFS/ME’, ‘ME/CFS’ in combination with ‘virome’, ‘microbiome’, ‘metabolome’, ‘metagenomics’, ‘viromics’ and ‘metabolomics’. The resulting papers were screened according to abstract contents. Articles were excluded if an intervention was used (e.g. probiotics) and measurements not reported prior to the invention. This resulted in 11 papers that had examined the microbiome and/or intestinal metabolome of ME/CFS patients, dating from 1998 to 2017 (Table 2). Due to inconsistencies in study design including small sample sizes, different sequencing platforms and bioinformatics software analyses, microbial sequencing depth and a single time point‘snapshot’ of sampling and analysis; it was not possible to compare the studies statistically. However, from examining the articles individually there is sufficient evidence to support the claim of an altered intestinal microbiome in ME/CFS patients.

**Table 2:** ME/CFS microbiome articles selected following literature review

Number	Year	Author	Title	Area of study
1	2017	Armstrong [93]	The association of faecal microbiota and faecal, blood, serum and urine metabolites in ME/CFS	Microbiome and metabolites
2	2017	Nagy-Szakal [90]	Faecal metagenomic profiles in subgroups of patients with ME/CFS	Microbiome
3	2016	Giloteaux [91]	Reduced diversity and altered composition of the gut microbiota in individuals with ME/CFS	Microbiome
4	2016	Giloteaux [107]	A pair of identical twins discordant for ME/CFS differ in physiological parameters and gut microbiome composition	Microbiome and virome
5	2013	Fremont [92]	High-throughput 16S rRNA gene sequencing reveals alterations of intestinal microbiota in ME/CFS patients	Microbiome
6	2009	Sheedy [94]	Increased d-lactic acid intestinal bacteria in patients with CFS	Microbiome and metabolites
7	2009	Fremont [109]	Detection of herpes virus and parvovirus B19 in gastric and intestinal mucosa of CFS patients	Virome
8	2008	Chia [108]	CFS is associated with chronic enterovirus infection of the stomach	Virome
9	2007	Evengård [176]	Patients with CFS have higher numbers of anaerobic bacteria in the intestine compared with healthy subjects	Microbiome
10	2001	Butt [177]	Bacterial colonosis’ in patients with persistent fatigue	Microbiome
11	1998	Butt [98]	Faecal microbial growth inhibition in chronic fatigue/pain patients	Microbiome and metabolites

Abbreviations: CFS, chronic fatigue syndrome; ME, myalgic encephalomyelitis

The literature search revealed nine articles that examined the microbiome in ME/CFS patients, with three articles also examining intestinal metabolites (Table 3); it is challenging to compare these studies because of different diagnostic criteria, patient selection, use or non-use of appropriately matched control subjects and microbial identification techniques. Of the nine articles, four used sequencing technologies, but different platforms, with five using culture-based techniques. One study (2017) performed metagenomic sequencing on 50 patient samples and was able to determine species-level differences compared with samples from control subjects [90]. A simple comparison between studies revealed eight similar results and seven conflicting results (Table 4). For example, while Giloteaux et al. [91] and Armstrong et al. [93] reported a general decrease in bacterial abundance, Sheedy et al. [94] reported an increase. These studies utilized different microbial identification techniques, which might account for the conflicting results. The lack of statistical analysis of the datasets constrains direct cross-study comparisons. From the limited cross-study analysis shown in Table 4, one finding of note was the decrease in *Faecalibacterium* seen in three studies. A reduction in butyrate-producing genus, which includes *Faecalibacteria* has been associated with dysbiosis in CD patients [95]. Butyrate has several protective properties, including improving the mucosal barrier, and immunomodulatory and anti-inflammatory effects by down-regulating pro-inflammatory cytokines [96]. However, decrease in the relative abundance of *Faecalibacterium* are associated with several other disorders and is not therefore specific for ME/CFS [97]. Interestingly, there was an increase in Enterobacteriaceae in two studies [91,98]. However, this may result from ME/CFS symptoms instead of a disease-specific microbial alteration. Enterobacteriaceae are dominant in the upper GI tract and are present in at low levels in the faeces of healthy individuals [99]. These taxa likely become enriched with faster stool transit time (i.e. signature of diarrhoea). The notable increase in this family would be consistent with increased transit time and reported in IBS-like symptoms in patients [100]. A depletion in the butyrate-producing family Ruminococcaceae was recorded in two studies and is also associated with diarrhoea [101]. Interestingly, *Bacteroides* spp. known for producing butyrate were reduced in several studies [97]. It would be beneficial to perform longitudinal studies of the microbiome in ME/CFS patients throughout the duration of the illness. This may provide more insightful clues as to the significance of any microbiome compositional changes with disease progression and severity within an individual patient. Several studies have been published on the longitudinal evaluation of ME/CFS patients; however, these focus on immune aspects, rehabilitative treatments and employment status [102–105].

**Table 3:** Overview of articles selected studying the microbiome in ME/CFS

Author	Number of patients	Number of controls	Studying	Study design
Armstrong [93]	34	25	Microbiome and metabolites	Culture + MS
Nagy-Szakal [90]	50	50	Microbiome	Metagenomics
Giloteaux [91]	48	39	Microbiome	16s rRNA gene sequencing
Giloteaux [107]	1	1	Microbiome and virome	16s rRNA gene sequencing
Fremont [92]	43	36	Microbiome	16s rRNA gene sequencing
Sheedy [94]	108	177	Microbiome and metabolites	Culture
Evengard [176]	10	10	Microbiome	Culture
Butt [177]	1390	-	Microbiome	Culture
Butt [98]	27	4	Microbiome and metabolites	Culture

Abbreviation: MS, mass spectrometry

**Table 4:** Basic comparison of microbiome composition alterations noted in articles selected for review

	Article details								
Microbiome comparison	Armstrong (2017) [93]	Nagy-Szakal (2017) [90]	Giloteaux (2016) [91]	Giloteaux (2016) [107]	Fremont (2013) [92]	Sheedy (2009) [94]	Evangård (2007) [176]	Butt (2001) [177]	Butt (1998) [98]
Overall abundance	↓		↓			↑			
Phylum Firmicutes			↓	↑					
Phylum Proteobacteria			↑	↓					
Family Bacteroidaceae			↓	↑					
Family Enterobacteriacaeae			↑						↑
Family Prevotellaceae			↑	↓					
Family Rickenellaceae			↓	↓					
Family Ruminococcaceae			↓	↓					
Genus *Bacteroides*	↓								↓
Genus *Bifidobacterium*			↓	↓			↑	↓	↓
Genus *Clostridium*		↑	↓						
Genus *Coprobacillus*		↑	↑						
Genus *Faecalibacterium*		↓	↓	↓					
Genus *Haemophilus*		↓	↓						
Genus *Ruminococcus*			↓		↓				
Species *Enterococcus faecalis*						↑			↑
Species *E. coli*								↓	↓

Seventeen criteria were either similar or conflicting between studies (microbiome composition). The down arrows represent a decrease in patients and up arrows represent an increase in patients.

As with any microbiome study, it is difficult to determine if the alterations observed cause, precede or correlate with disease. The microbiome of a patient would exhibit disease-specific microbial signatures and general microbial changes due to an unbalanced microbiome [106]. It is important, therefore, to separate microbial alterations associated with an unbalanced microbiome from those associated with a specific disease (microbiome disease biomarkers).

### Virome and ME/CFS

Of the 11 articles selected in our literature search, only 3 examined the intestinal virome of CFS/ME patients (Table 5). Of these, two articles used direct virus detection (e.g. PCR or immunostaining) and one article used a high-throughput sequencing technique (Illumina MiSeq). An increase in bacteriophage richness, particularly Siphoviridae and Myoviridae, in patients was noted in the Giloteaux et al. study [107]. However, this study is statistically underpowered due to its small sample size. Chia and Chia [108] and Frémont et al. [109] used virus detection techniques to examine the presence of eukaryotic viruses within the gastric/intestinal mucosa. These studies reported an increase in parvovirus B19, enteroviral RNA and viral capsid protein 1 in patients. Also of note, Nagy-Szakal et al. [90] used metagenomic based approach on a large (*n*=50) cohort of patients although they did not perform virome analysis on the dataset. The authors reported significant changes in the bacterial components of the microbiome in ME/CFS patients compared with controls [90]. Virome analysis could be performed with the available date to determine if significant changes are observed in the viral components of ME/CFS patients.

**Table 5:** Overview of articles selected studying the virome in ME/CFS

Author	Number of patients	Number of controls	Study design
Giloteaux [107]	1	1	Viral metagenomics
Fremont [109]	48	35	PCR detection
Chia [108]	165	34	PCR detection and immunoperoxidase staning

### Metabolomics studies

The identification of the bacterial and viral components of the microbiome is an important step forward, as is understanding how the use of nutrients by these microorganisms influences the overall metabolism within the gut. Metabolomics can be used to identify metabolites within the microbiome [110]. Only a handful of studies have attempted to characterize faecal metabolites in ME/CFS patients despite its potential for deciphering microbiome function (Table 6) [93,94,98]. There are significant challenges associated with identifying faecal metabolites due to differing metabolite properties and range of metabolite concentrations in samples [162,163]. A major challenge is not only to identify all metabolites (insufficient reference libraries available) but also to produce metadata (i.e. sample origin, tissue, experimental conditions) in a format that is easily interpreted [166]. The biological interpretation of metabolites as potential disease-associated biomarkers is often challenging as it requires data analysis and integration [167] and targeted and non-targeted metabolomics to dissect the metabolic pathway(s) and origin of metabolite(s) of interest [164]. Currently, ^1^H NMR is the most used analytical technique for metabolite profiling and is routinely used in clinical or pharmaceutical research and applications [165].

**Table 6:** Overview of articles selected for studying the metabolome in ME/CFS

Author	Number of patients	Number of controls	Study design
Armstrong [93]	34	25	NMR spectroscopy
Sheedy [94]	108	177	C^13^-labelled bacteria/metabolites for HPLC and NMR
Butt [98]	27	4	Specific metabolites

Abbreviations: NMR, nuclear magnetic resonance; HPLC, high performance liquid chromatography

Armstrong et al. [93] quantified metabolites using high-throughput ^1^H NMR spectroscopy from ME patient faecal filtrates. This technique provides a non-targeted metabolic profile that measures all high concentration metabolites with non-exchangeable protons [111]. In addition to faecal metabolomics, the authors performed urine and blood serum metabolite analysis. The present study presented a robust metabolome workflow and eluded to the relationship of faecal metabolites and microbes with host blood serum and urine metabolites [93]. Two older studies used selective culture based systems to examine the metabolic output of specific bacteria [94,98]. It is difficult to draw conclusions from these older studies because of the culture-based techniques used. It is possible that the isolation of bacteria from the complex intestinal environment alters the excreted/secreted metabolites, resulting in metabolites specific to the artificial *in vitro* culture environment. To obtain a true picture of the faecal metabolome, samples should be prepared directly from the faecal sample.

Unfortunately, it is not possible to compare these three metabolomics studies directly because different metabolites were studied. However, it is possible to make general comparisons between metabolites and microbes. For example, Sheedy et al. [94] reported an increase in lactic acid and an increase in *Enterococcus faecalis*, a lactic acid producing bacteria. Interestingly, Armstrong et al. [93] reported a general increase in the short chain fatty acids (SCFAs) butyrate, isovalerate and valerate. This contradicts the microbiome studies as known SCFA-producing bacteria (*Faecalibacterium, Eubacteria, Roseburia* and *Ruminococcus*) were consistently decreased across multiple studies [90–92,107]. A decrease in lactate was also reported in this study [93]. Several bacterial members of the microbiota produce lactate, which is the most common short chain hydroxyl-fatty acid in the intestinal lumen [97,112]. It can be converted into other SCFAs by a subgroup of lactate-fermenting bacterial species. Changes in these lactate-fermenting bacterial species were not noted in the current microbiome studies. Future studies will need to examine microbiome and metabolome alterations in tandem and then integrate the data to reveal a truer picture of microbiome metabolism.

## Studying the microbiome: techniques and challenges

**Figure 3:**
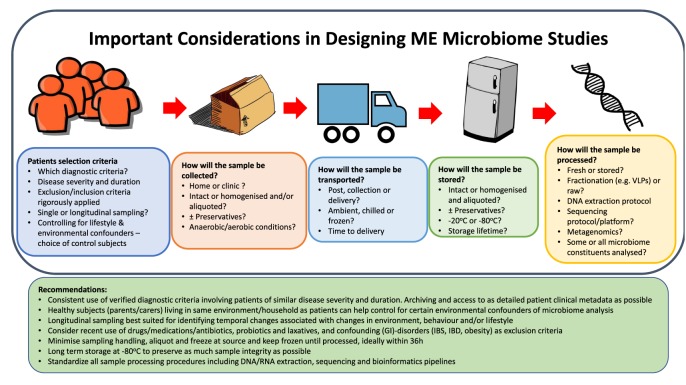
Important considerations in designing ME microbiome studies Recommendations for designing a microbiome study and important questions to consider

Within recent years, the increased interest in trying to understand the effect of the microbiome on health and disease has resulted in significant advancements in techniques to characterize it [9–11]. In particular, metagenomics is increasingly popular and favoured over sequencing bacterial 16s rRNA due to increased taxonomic sensitivity and potential for functional interpretation. Additionally, established techniques are being applied to microbiome research, such as metabolomics [11,113]. The research at the Quadram Institute Bioscience is focused on optimizing protocols, standardizing microbiome studies and applying this to ME/CFS. There are several pitfalls and challenges associated with microbiome studies, which need to be addressed prior to patient recruitment and sample collection [114]. The considerations that need to be made in designing microbiome studies in ME/CFS and some recommendations are outlined in Figure 3. Below we describe in some detail the particular constraints on microbiome and virome studies in ME/CFS and the approaches that can be taken to mitigate against or overcome them.

### Patient recruitment

A standardized criterion for ME/CFS diagnosis is lacking, with diagnosis relying heavily upon clinical observations and exclusion [83,115]. Multiple diagnosis checklists have been created, with each checklist differing slightly on symptom emphasis and severity [69,83,84]. The International ME criteria and Canadian criteria place greater emphasis on the delayed exacerbation of physical and mental symptoms following exertion. However, it does not exclude psychiatric illness such as depression or anxiety [116,117]. Therefore, it is difficult to determine how many patients recruited have an accurate diagnosis of ME/CFS and how many have been misdiagnosed using inadequate criteria. Several studies have attempted to address this by using multiple diagnostic checklists, with the majority using the 1994 Fukuda diagnostic scale. The severity of ME/CFS is ranked according to impact of illness upon daily life and ranges from mild to very severe. The severity grade given to a patient is subjective and generally given by the diagnosing clinician. Several studies have used patient questionnaires to assess the level of illness [90,91,94,107]. However, four different patient questionnaires were used by four different studies (Short Form 36 Healthy Survey, Multidimensional Fatigue Inventory, Bell’s Disability Scale and McGregor 1995 questionnaire). Due to the multifactorial nature of the disease, standardization in diagnosis and disease severity are imperatives. These are a basic requirement to produce robust and reproducible microbiome studies. It is very difficult to determine if any microbiome differences are due to a true ME/CFS signature or complexities of patient recruitment. Future studies should aim to stratify patients according to disease duration and onset (sudden or gradual).

As microbiome research has increased, the need for properly matched controls has become apparent. The complexity of the GI microbiome and potential role within healthy/diseased states produce confounding factors [118]. As many of these confounding factors need to be taken into consideration in a microbiome study, and as many as possible should be accounted for or eliminated. Age, lifestyle, medications and drug use, geography and diet have all been reported to influence microbiome function and composition [5–7]. The effect of antibiotics on the microbiome is well documented [119]. However, other prescription and recreational drugs can affect microbiome analyses [120]. For example, decreasing stomach acidity with proton pump inhibitors allows upper GI microbes to move down into the intestine more readily, altering the composition of the lower GI microbiota and increasing risk of *C. difficile* infections [121].

Diet also influences the microbiome. Long-term dietary patterns have been linked to faecal microbiomes dominated by certain genera [118,122]. High protein/animal fat diets are associated with the prevalence of *Bacteriodes*, whereas diets high in carbohydrates are associated with high *Prevotella* [123]*.* To account for this, details of food consumption at least 48 h prior to sample collection should be obtained. Moreover, healthy household controls could be used to identify and exclude environmental confounders (e.g. diet, living environment); increasing the likelihood of identifying disease-specific microbiome signatures [118]. Additionally, the microbiome changes during aging and declines in diversity in the elderly [124]. Therefore, the use of age-matched controls would be beneficial to account for this important variable.

Recently, the influence of gender on the microbiome (termed as ‘microgenderome’) has become evident [125–128]. The intestine and its’ microbiome serves as a virtual endocrine organ due to the metabolites and neurotransmitters and hormones it can produce [129]. For example, early microbial exposure increases testosterone levels in male mice, leading to a protective effect against T1D [128]. Additionally, microbiome alterations are observed in pre- and post-menopausal women; highlighting hormonal cross-talk within the microbiome [130]. Certain microbes have also been discovered to be a source of hormones and neurotransmitters. Experimental models have confirmed the bidirectional relationship between the intestinal microbiota, sex hormones and the immune system and provided an explanation for sexual dimorphism in T1D [128,131]. The results of these studies revealed evidence of sex-specific microbial communities, sex-specific responses to the same microbial communities, the role of sexual maturation impacting on changes on microbial communities and that microbial communities can play a protective and therapeutic role by influencing hormonal, metabolic and immune pathways [125–128]. A 2015 study compared the microbiome of male and female patients with ME/CFS revealing significant sex-specific interactions between Firmicutes (*Clostridium, Streptococcus, Lactobacillus and Enterococcus*) and symptoms, regardless of compositional similarity of microbial levels across the sexes [132]. This study highlights the need for gender-matched controls to account for any gender bias from future microbiome studies.

Although it is often impractical and perhaps impossible to control for all confounding factors within a microbiome study, efforts should be made to account for as many as possible.

### Sample collection, storage and processing

As the number of microbiome studies has increased, the need for consistency in sampling techniques and standard operating procedures (SOPs) has also increased. An excellent review of the critical factors for sample collection, storage, transport and ‘gold standard’ techniques for longitudinal microbiome studies in human populations was recently published [114]. The most important considerations for storing microbiome samples are to reduce changes in the original microbiota from sample collection to processing and to keep storage conditions consistent for all samples in a study [133,134]. Sample storage conditions are not always consistent due to study or research group-specific downstream applications and resource limitations. Additionally, considerations are not always taken for preserving anaerobic bacteria within an anaerobic environment. Different studies often store samples at differing temperature (e.g. 4 to −80°C), affecting the long-term preservation of certain bacteria [114]. Additionally, the length of time for which the sample is stored and frequency of freeze/thaw cycles can significantly affect the microbiome composition. For example, *Bacteroides* is sensitive to freezing and should be processed within 6 weeks of storage (at −80°C) to avoid bacterial degradation [135,136]. The microbiome and ME/CFS studies reviewed here used different sample collection and storage techniques; including storage at <12°C, immediate processing, −20 and −80°C. For logistical reasons, it can be difficult to standardize this across all studies. However, it is important to be aware of these limitations.

The microbe composition changes laterally and longitudinally along the GI tract, therefore it has been suggested that there is significant variation within a single faecal sample. A 2015 study reported a reduction in intrasample variation following homogenization of the whole faecal sample [137]. However, several studies use a random section of the faeces without homogenization.

Additionally, different DNA extraction techniques have been used as a prelude to sequencing (MoBio PowerSoil DNA isolation kit, QIAmp DNA Stool Mini Kit and DNeasy Blood and Tissue kit). These kits differ in protocol, bead size, reagents used and are likely to introduce unnecessary bias [138].

### Identification of prokaryotes

The development of sequencing, characterization of the bacterial component of the faecal microbiome relied on culture-based techniques that allow the identification of anaerobic and aerobic bacteria using selective or non-selective culture conditions and media; albeit taxonomic resolution and sensitivity is relatively low [12]. However, this approach does inform the cultured organism’s growth requirements and substrate utilization and other physiological parameters, which cannot be obtained from sequence-based approaches [12]. Next-generation sequencing technology now makes it possible to characterize the bacterial microbiome using the 16S rRNA gene ‘fingerprint’ for identification and as an indicator of genetic diversity [4]. The 16S rRNA gene was chosen because of its relatively small size (~1.5 kb) and harbouring enough variation to distinguish between different species, yet enough similarity to assign members belonging to the same larger phylogenetic group (e.g. order, family or phylum) [5,139]. However, this approach has its limitations. It only detects and analyses a short, specific genomic region and taxonomic resolution or functional inference is therefore limited [11]. For example, this assay cannot recognize the different serovars within *Salmonella enterica* or detect toxin genes that could distinguish pathogenic *C. difficle* or distinguish pathogenic *Escherichia* strains from non-pathogenic strains [140]. This is particularly problematic in comparative studies of the microbiome in healthy and diseased states. It also provides no insight into functionality of the bacteriome [11].

Metagenomic sequencing is increasingly being chosen over 16S rRNA sequencing due to its higher taxonomic resolution and ability to infer functional potential [140]. It provides sequence information from the collective genomes of the microbiota, which in turn can be used to infer or predict functional contributions and biological roles of this complex community in human health and disease [11,139]. However, the absence of whole genome sequences in public databases limits the ability to identify gene function based on known sequence information. In comparison with 16S-based sequencing approaches, whole community metagenomics with an appropriate sequencing depth and coverage can be used to identify other microbes (i.e. archaea and viruses) within the microbiome [140,141]. Although it is possible to infer functional potential from metagenomic analysis through gene presence/abundance, the presence of a gene does not necessarily infer function; it is possible for the gene to be present but not transcribed [113]. Therefore, careful consideration needs to be taken when inferring functional potential from metagenomic sequences and, if possible, the predicted function should be examined using laboratory based techniques (e.g. antibiotic resistance), assuming the candidate microbe(s) can be cultured in isolation.

To date, only one study has utilized metagenomics in ME/CFS microbiome studies [90]. However, the analysis was incomplete and did not fully exploit the data produced. Whenever possible, metagenomics should be applied to microbiome studies in ME/CFS in order to achieve the required taxonomic resolution to fully examine the bacteriome and virome.

### Identification of viruses

Virus genomes do not encode universally conserved genes such as the 16S or 18S genes of prokaryotes and eukaryotes respectively, and they are genetically highly diverse [142]. Consequently, it is not possible to use metataxonomic approaches such as 16S rRNA gene sequencing to characterize VLPs within an ecosystem [20]. Traditionally, classical approaches of microscopy and cultivation have been used to characterize VLPs isolated from faecal samples originating in the human intestine [143,144]. The only reliable molecular method currently available for surveying the human virome is metagenomics. However, to achieve an adequate sequencing depth, lytic VLPs need to be separated from the faecal material [145,146]. An excellent review describing the human virome and its characterization was recently published [147].

The efficient isolation of VLPs is an essential step in viral metagenomics in order to obtain an accurate picture and profile of the virome [148,149]. The workflow for sequencing the nucleic acid in VLPs (Figure 2) from faecal material begins with homogenization of faeces in buffer, centrifugation to remove cell debris followed by filtration to remove bacteria [150]. Ultracentrifugation can be used to separate the sample into differing densities and a specific density containing VLPs can be selected for downstream processing. Within the intestinal microbiome community, viral genomes represent a small proportion of the total DNA compared with bacterial genomes [149,151]. It is important therefore to use a reliable, robust and efficient VLP isolation protocol with as few manipulations of the sample as possible to minimize loss of VLPs. Various VLP protocols have been published that differ in details such as filter pore size, centrifugation speed and the inclusion/omission of ultracentrifugation [148–150,152,153]. Importantly, these protocols have yet to be directly compared. It is highly desirable therefore that standardized faecal VLP isolation and DNA extraction techniques are adopted to enable direct comparisons of datasets from different virome studies.

Viral metagenomic data are analysed in a manner similar to bacterial metagenomic data [154,155]. High-quality reads are aligned to reference databases, assembly is then attempted with non-aligned reads and functional characteristics inferred [147]. However, the lack of conserved genes, high genetic variation and under-representation of virus genomes within reference databases means a minority of the reads can be taxonomically assigned. It is predicted that less than 0.001% of the predicted phage diversity is represented in global sequence databanks [156]. One virome study has reported that 98% of the generated reads did not significantly match to an identified sequence within a database [157]. Therefore, a majority of sequencing reads are unassigned to any known genomes and are considered ‘viral dark matter’. Additionally, assembly of sequencing reads is made difficult due to their short-read lengths [158,159]. Several research groups are now investigating the possibility of long-read sequencing to characterize the virome. The release of the Oxford Nanopore MinION has drastically reduced the cost of long-read sequencing (compared with PacBio sequencing). These have the potential to provide complete or near complete phage genomes without the need for alignment or representation in databases [160].

Perhaps the biggest challenge in studying the intestinal virome is the lack of bioinformatics tools for the analysis of sequence data [146,147,161]. To date, there is no easy-to-use pipeline that uses raw reads, can remove host DNA, can search for bacterial contaminants and assign taxonomy and functionality to viruses within the sample. However, efforts are being made to generate such tools. In addition to isolation and sequencing of VLPs, it is possible to identify prophages and the bacterial host(s) from metagenomic sequencing. To accurately study the virome, both techniques should be utilized to study the lytic and lysogenic phages [154,155,157]. The Norwich U.K., ME/CFS research group is currently optimizing and standardizing VLP isolation and DNA/RNA sequencing protocols in addition to developing fit-for purpose viromics pipelines to comprehensively analyse the virome in ME/CFS patients.

## Concluding remarks

Several microbiome studies have been performed on ME/CFS patients in the hope of identifying disease-specific signatures. This disease should be viewed as multifactorial and that the alteration of one body system (e.g. microbiome) may not be the exclusive cause. The dysregulation of the microbiome may be variously placed in a disease progression pathway interfacing with other systems (immune, neuroendocrine and mitochondrial), tipping the body into persistent imbalance. Although studies to date often report conflicting results, microbiome dysbiosis in ME/CFS patients is evident. However, in order to discover disease-specific microbe alterations, future studies need to adopt standardized techniques and analyses. The recent advancements in sequencing technology allows the characterization of the previously neglected virome. As virome research increases, it is becoming clear that the virome can directly and indirectly affect host health, and may play a role in the pathogenesis of ME/CFS. Confirmation of such a role will be largely dependent on the adoption of robust patient selection, reproducible study design and appropriate data analyses by different research groups investigating the microbiome/virome in complex diseases such as ME/CFS.

## Clinical perspectives

- Several studies have reported alterations in the intestinal microbiome of ME/CFS patients, suggesting the involvement of microbial dysbiosis.
- Study design needs to be consistent to allow statistical comparison between different microbiome studies.
- Future microbiome studies should take account of the virome.

## Abbreviations

CD: Crohn’s disease
CFS: chronic fatigue syndrome
GI: gastrointestinal
IBD: inflammatory bowel disease
IBS: irritable bowel syndrome
IL: Interleukin
ME: myalgic encephalomyelitis
ME/CFS: myalgic encephalomyelitis/chronic fatigue syndrome
SCFA: short chain fatty acid
T1D: type 1 diabetes
TNFα: Tumor necrosis factor alpha
VLP: virus-like particle
WHO: World Health Organization
